# Scoring of senescence signalling in multiple human tumour gene expression datasets, identification of a correlation between senescence score and drug toxicity in the NCI60 panel and a pro-inflammatory signature correlating with survival advantage in peritoneal mesothelioma

**DOI:** 10.1186/1471-2164-11-532

**Published:** 2010-10-01

**Authors:** Kyle Lafferty-Whyte, Alan Bilsland, Claire J Cairney, Lorna Hanley, Nigel B Jamieson, Nadia Zaffaroni, Karin A Oien, Sharon Burns, Jon Roffey, Susan M Boyd, W Nicol Keith

**Affiliations:** 1 Centre for Oncology and Applied Pharmacology, University of Glasgow, Cancer Research UK Beatson Laboratories, Garscube Estate, Switchback Road, Bearsden, Glasgow G61 1BD, UK; 2 Molecular Pharmacology Unit, Department of Experimental Oncology and Laboratories, Fondazione IRCCS Istituto Nazionale dei Tumori, Via Venezian, 1, 20133 Milan, Italy; 3 Cancer Research Technology Limited, Wolfson Institute for Biomedical research, Gower Street, London, WC1E 6BT, UK; 4 CompChem Solutions, St John's Innovation Centre, Cowley Road, Cambridge, CB4 0WS, UK

## Abstract

**Background:**

Cellular senescence is a major barrier to tumour progression, though its role in pathogenesis of cancer and other diseases is poorly understood in vivo. Improved understanding of the degree to which latent senescence signalling persists in tumours might identify intervention strategies to provoke "accelerated senescence" responses as a therapeutic outcome. Senescence involves convergence of multiple pathways and requires ongoing dynamic signalling throughout its establishment and maintenance. Recent discovery of several new markers allows for an expression profiling approach to study specific senescence phenotypes in relevant tissue samples. We adopted a "senescence scoring" methodology based on expression profiles of multiple senescence markers to examine the degree to which signals of damage-associated or secretory senescence persist in various human tumours.

**Results:**

We first show that scoring captures differential induction of damage or inflammatory pathways in a series of public datasets involving radiotherapy of colon adenocarcinoma, chemotherapy of breast cancer cells, replicative senescence of mesenchymal stem cells, and progression of melanoma. We extended these results to investigate correlations between senescence score and growth inhibition in response to ~1500 compounds in the NCI60 panel. Scoring of our own mesenchymal tumour dataset highlighted differential expression of secretory signalling pathways between distinct subgroups of MPNST, liposarcomas and peritoneal mesothelioma. Furthermore, a pro-inflammatory signature yielded by hierarchical clustering of secretory markers showed prognostic significance in mesothelioma.

**Conclusions:**

We find that "senescence scoring" accurately reports senescence signalling in a variety of situations where senescence would be expected to occur and highlights differential expression of damage associated and secretory senescence pathways in a context-dependent manner.

## Background

Based on observations in ageing cultured normal cells, cellular senescence has traditionally been regarded as a permanent cell cycle arrest state which presents a major barrier to uncontrolled cellular proliferation and tumour development [1]. The role of telomeres in initiation of physiological replicative senescence in such cells is now firmly established, in which failure of the protective cap Shelterin on highly shortened structurally dysfunctional telomeres causes direct engagement of the DNA damage signalling machinery [2]. In addition to division-associated telomere attrition, several other stimuli provoke a rapid senescence response including DNA damaging insults and supra-physiological expression of oncogenes [3,4]. To acquire a fully transformed phenotype, cancer cells must bypass senescence induced by such stimuli and continue to proliferate, commonly by inactivation of core sentinels of cellular stress such as p53 and pRb [5]. An interesting corollary of findings that appropriate molecularly targeted interventions are able to bypass and even reverse established senescence, suggest that the phenotype is likely to be considerably more plastic and its control more dynamic than previously envisaged [6,7].

In the context of cancer therapy, it is now clear that both radiotherapy and chemotherapeutic agents provoke a rapid cell cycle arrest response termed "accelerated senescence". Seminal observations from an apoptosis deficient mouse model indicated that senescence contributes to anti-tumour efficacy of cyclophosphamide [8], and many other cytotoxic agents have been found to elicit accelerated senescence in cancer cells at substantially lower doses than those required to promote apoptosis. Therefore, despite inactivation of some key pathways, many tumour cells retain the ability to exit the cell cycle under appropriate treatments. Although physiological and accelerated senescence share many morphological and molecular similarities, some potentially important differences between the phenotypes have been observed such as differential regulation of methylation control pathways [9]. Therefore understanding both the mechanisms behind senescence in different cell and tissue types in response to different agents and the activity of those pathways in vivo is of growing importance.

For some years, detection of senescence in tissue culture systems relied primarily on phenotypic changes such as morphological alterations and β-galactosidase staining [10] with a few well established molecular markers such as p16 and p21 expression. However, evidence is accumulating to suggest that senescence should not be regarded exclusively in terms of the activity a small number of molecules. Rather, it is perhaps best viewed as a larger signalling pathway or a new ontology, wherein complex and tightly regulated gene expression programmes integrate diverse cell-autonomous and non-autonomous processes. Recent studies have both improved the mechanistic understanding of senescence and provided new molecular markers through identification of phenomena such as telomere induced DNA damage foci, senescence associated heterochromatin foci and the senescence associated secretory phenotype [11-13]. Availability of these new markers provides an opportunity for a "pathway-directed" expression profiling approach in relevant tissues using multi-gene signatures relating to divergent aspects of senescence signalling.

In this study we used a DNA damage associated senescence (DAS) signature and a modified secretory senescence (mSS) signature compiled from recent data on senescence associated gene expression [11,12,14-23] to probe publicly available tumour gene expression data sets and our own mesenchymal tumour set using a scoring approach based on relative expression of pro- and anti-senescence markers. We first validated the approach using public data sets involving interventions in which increased senescence would be expected. The sets chosen for this analysis examine radiotherapy of colon adenocarcinoma [24], doxorubicin and 5-FU treatment of breast cancer cell lines [25], and replicative senescence of mesenchymal stem cells [26]. In each case, we find that the scoring approach does indeed report increased pro-senescence signalling and allows dissection of the relative contributions of DAS and mSS subtypes. In another public dataset [27] we found that senescence signalling increases during melanoma progression, presumably reflecting multi-step bypass of tumour suppression programmes.

Having validated the approach in several situations in which senescence is expected we investigated correlations between senescence score and growth inhibition in response to ~1500 compounds screened for toxicity against the NCI60 panel. We examined relationships between DAS or mSS expression and compound sensitivities and, through chemoinformatic prediction of compound activities, we investigated the representation of pharmacophores against several broad protein target classes in the significant DAS or mSS related compound set. Finally, we performed an in-depth examination of the mSS signature in our own mesenchymal tumour typeset comprising 24 liposarcomas, 16 malignant peripheral nerve sheath tumours (MPNST) and 20 peritoneal mesotheliomas [28]. Through the use of hierarchical clustering we examined expression patterns and resulting tumour groupings in relation to histological characteristics and outcome.

## Results

### Development of senescence scoring approach

**Figure 1:**
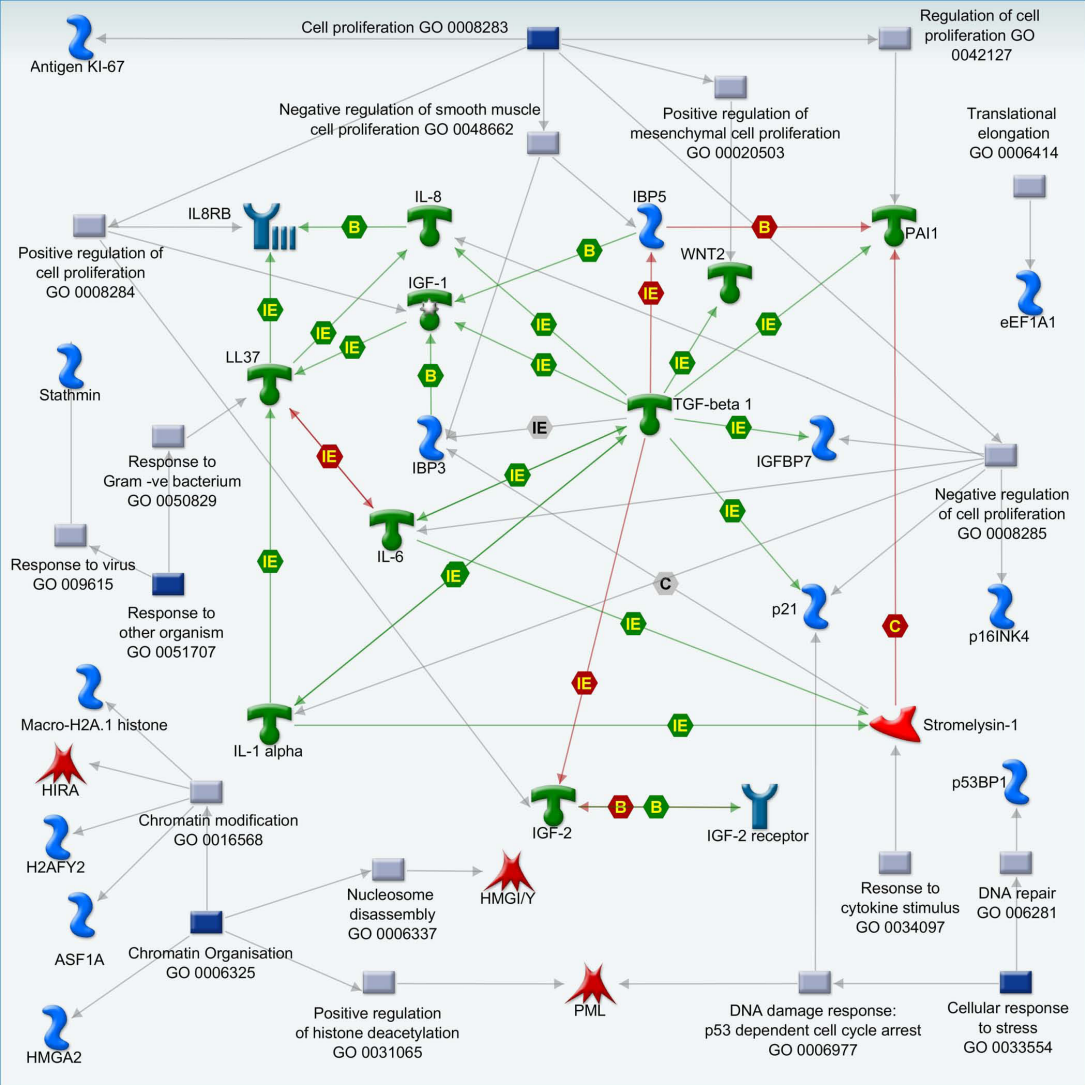
**Representative Gene Ontology associations and functional interactions between genes in the senescence scoring signature**. The figure illustrates the cooperation between diverse processes including cell proliferation, chromatin remodelling and stress/inflammatory responses in the senescence phenotype. Blue and grey boxes represent parent and child GO processes, respectively. Red and green arrows represent negative and positive regulation, respectively. Mechanism: IE, influence on expression; B, binding; C, cleavage.

Bypass of senescence is a requirement for full transformation. However, many cancer cells retain the ability to undergo senescence in response to genotoxic insults [3,4]. Therefore, tumours must retain extant senescence pathways, though it is unclear how their expression is changed during the process of transformation in different cells and whether these expression levels might relate to the propensity to undergo cell cycle arrest in response to appropriate therapeutic intervention. In this study we have explored the use of a senescence scoring approach based on expression profiling of well-defined senescence markers as a means to evaluate latent senescence pathways in relevant samples. Published literature was mined for gene expression signatures and biomarkers of senescence (Table 1) and two sub-signatures were established. DAS biomarkers represent genes with known roles in senescence from literature involving DNA damage and chromatin responses [29-31]. Additionally, a modified secretory senescence (mSS) signature was created by combination of senescence messaging secretome/senescence associated secretory phenotype (SMS/SASP) signatures with a signature of 4 genes representing DNA damage and telomere dysfunction which increase in expression/activity in senescent cells and are detectable in serum of ageing human patients [11,12,14,15]. At present our measure of the senescence response relies on relative expression of individual biomarkers. We propose that with an ever increasing number of genes associated with the senescence response it should be viewed as a new ontological process consisting of a complex network of gene expression and signalling pathways. To demonstrate this point Figure 1 shows known functional relations between the genes in Table 1, which are present in the MetaCore interactions database from GeneGo Inc [32]. Additionally, the figure shows representative GO processes for each gene, which further illustrates the cooperation of diverse processes of damage and inflammation in senescence.

**Table 1:** Gene expression changes and senescence signatures.

Originating Senescence Signatures	Genes	Expression Direction in Senescence
	p16	Increase
	HMGA1	Increase
	HMGA2	Increase
	HIRA	Increase
	ASF1a	Increase
**DAS Biomarkers**	H2AFY	Increase
[16,17,19-23]	H2AFY2	Increase
	PML	Increase
	MMP3	Increase
	53BP1	Increase
	p21	Increase
	Ki67	Decrease

	IL6	Increase
	IL8	Increase
	PAI1	Increase
	IGFBP3	Increase
	IGFBP5	Increase
	IGFBP7	Increase
	IL1A	Increase
**mSS Biomarkers**	CXCR2	Increase
[11,12,14,15]	IGF1	Decrease
	IGF2	Decrease
	WNT2	Decrease
	CAMP	Increase
	STMN1	Increase
	EEF1A1	Increase
	IGF2R	Increase
	TGFB1	Increase

**Figure 2:**
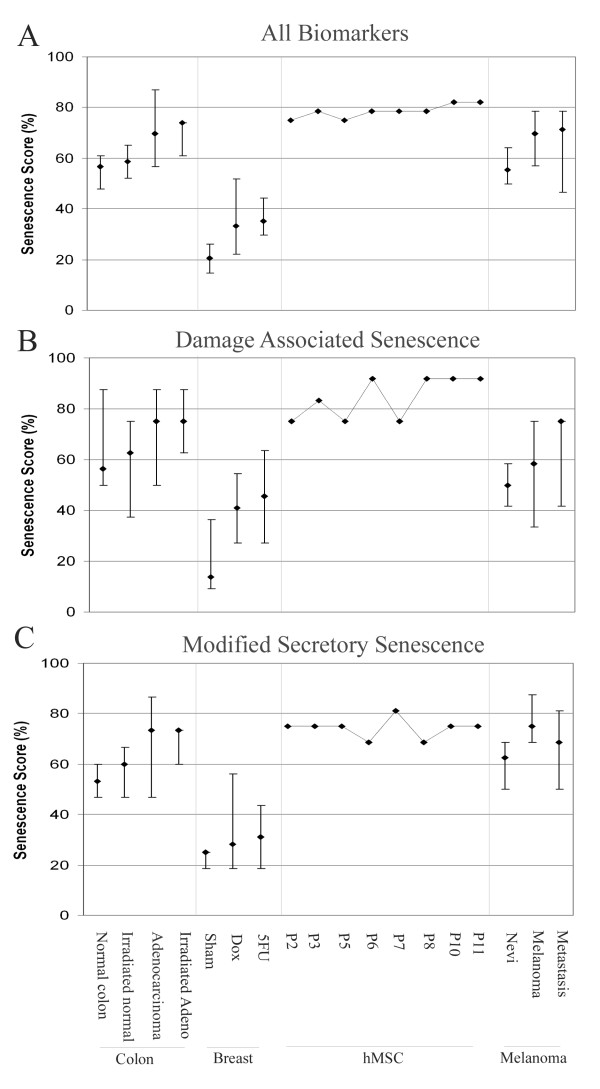
**Senescence scoring of public data sets**. Datasets tested were: irradiated and non-irradiated colorectal tumour and normal tissue (GSE15781); doxorubicin and 5-FU treated breast cancer cell lines (GSE1647); mesenchymal stem cells cultured to replicative senescence (GSE9593); a melanoma progression set (GSE4587). All sets were scored using (A) All markers from Table 1, (B) DAS markers, (C) mSS markers. Median senescence scores are shown with bars representative of the maximum and minimum values. In the case of the hMSC set, the time-course data are derived from the cells of a single donor without replication and therefore, no bars are shown.

To assign senescence scores, microarray data was imported into BRB ArrayTools [33] and filtered to show only those genes associated with senescence. Data was normalised to a median value across all arrays to make all data relative to a median value of 1. Therefore genes with expression greater than 1 were up-regulated and less than 1 down-regulated. Each gene was scored as senescent or not depending on whether expression was in a pro-senescence direction (see Table 1) with reference to the median expression level. For example, expression of the proliferation-associated gene *KI67 *below median would be marked "senescent" but above median expression would not. The number of genes marked "senescent" was then calculated for each sample and expressed as a percentage of the total number of genes expressed in each particular signature, all biomarkers in Table 1, DAS biomarkers only and mSS biomarkers only, to give an overall senescence score for each signature. By this approach, we hypothesised that the level of pro-senescence signalling in each pathway can be measured. Although very simple mathematically, taking the percentage of genes still signalling in a senescent manner provides a single continuous senescence measurement in human tumours incorporating the sum contributions of many pathways, something which has not previously been reported. In addition the scoring workflow could be used as a hypothesis driving tool to investigate the effect of modulating the expression of different genes in the senescence signalling network or indeed applied to other biological scenarios by integrating alternative expression signatures. We have utilised this scoring system to perform investigations of senescence signalling in various settings (Figure 2).

### Senescence signalling pathways induced by radiation therapy

Our first main aim was to establish proof of concept as to whether our scoring approach can provide a reliable readout in settings in which senescence would be predicted to occur. We therefore investigated whether senescence signalling was altered in tumours and normal tissue of the colon before and after radiotherapy in the publicly available dataset GSE15781 (Figure 2). Examination of all biomarkers showed that tumours had a higher level of senescence signalling compared to their respective normal tissues (score 69% and 56%, respectively) (Figure 2A). Furthermore, irradiated tumours have higher median levels of pro-senescence signalling than their non-irradiated counterparts (score: 74% in irradiated tumours compared with 69% in non-irradiated). These results suggest that the scoring system is able to capture increased senescence signalling resulting from radiotherapy.

As well as measuring overall senescence signalling, we assigned scores for the sub-signatures DAS and mSS. Irradiation of tumours causes high levels of DNA damage with the aim of causing cell necrosis or apoptosis [34]. Figure 2B shows that irradiated normal tissue has increased median DAS signalling compared to non-irradiated normal, however the overall spread of the data is lower in the irradiated normal tissue (50%-87% in non-irradiated compared to 37%-75% in irradiated normal). Median DAS signalling in adenocarcinoma is unchanged by irradiation, although the minimum scores are at a higher level in irradiated tumours than their non-irradiated counterparts (62% in irradiated compared to 50% in non-irradiated adenocarcinoma), suggesting a trend in favour of higher DAS signalling in irradiated tumours. Interestingly, irradiation did not increase mSS signalling in tumours (score: 73% pre- and post-irradiation). In contrast, normal tissues showed a median increase of 6.7% following irradiation (Figure 2C). Radiotherapy therefore appears to specifically activate the DNA damage and chromatin aspects of senescence whilst not altering levels of the secretory senescence signalling pathway in tumours. This finding may present novel opportunities for improved senescence induction through therapeutic activation of secretory pathways in these tumours.

### Senescence scoring in drug treated breast cancer cells

To assess the performance of the scoring approach in analysis of drug-induced accelerated senescence, we examined the public data set GSE1647 in which the authors investigated drug-specific toxicity-related expression profiles in a panel of breast cancer cell lines treated with chemotherapy drugs. Senescence was not directly assessed in this study, though each drug was administered at its IC50 for each cell line (assessed by a mitochondrial dye conversion assay). Because the set contains few replicates for each treatment, for statistical robustness we have here pooled data for the 3 cancer cell lines ZR-75-1, ME16C, and MCF7 treated for 24 hours with either doxorubicin or 5-FU, both of which have previously been observed to induce the accelerated senescence phenotype in tissue culture [35,36].

Across all cell lines, the median basal senescence score is comparatively low (20%). As observed with radiotherapy of colon adenocarcinoma, treatment with either 5-FU or doxorubicin results in induction of the overall senescence score (Figure 2A, 5-FU score: 35%; doxorubicin score: 33%). DAS markers have basal expression of 14% in untreated cells. As expected, these genes are strongly induced, resulting in median scores of 45.5% and 41% for 5-FU and doxorubicin, respectively (Figure 2B). The mSS component is also induced by 24 hour drug treatments, though more modestly than the DAS component, rising to 31% and 28% from a basal level of 25%. Thus, both radiotherapy and chemotherapy induce multiple senescence markers as expected, though mSS appears to have a greater contribution in the chemotherapeutic setting.

### Senescence signalling dynamics in human mesenchymal stem cells

Having shown that the scoring approach reports increased senescence signalling under therapeutically relevant conditions of genotoxic stress, we next investigated replicative senescence of human mesenchymal stem cells (hMSCs). We scored the senescence signalling signatures at passages 2 to 11 in hMSCs from the same patient (GSE9593). Prior to passage 11, at which time hMSCs were completely senescent, the authors of this study observed continuous changes in the cellular phenotype including gradual increases in cell size, autofluoresence, SA-bGal staining and osteogenic differentiation potential, while adipogenic differentiation potential slowly decreased [26]. These results are consistent with the model of partially stochastic dynamics in replicative senescence onset that has previously been proposed [37-39].

Our analysis of all markers (Figure 2A) is consistent with these observations, indicating that senescence gradually increases with passage. A plateau between passages 6 and 8 is evident when all markers are scored (score 79%). Strikingly, however, examination of the DAS and mSS signatures shows that this plateau is in fact a timed change in signalling type. Secretory senescence signalling remains level at 75% until passage 6 at which point a slight reduction to 69% is observed. At this time point there is a corresponding sharp increase in DAS signalling (75% to 92%). A subsequent spike in secretory (mSS) expression (score 81%) occurs at passage 7, followed by a return to base levels (Figure 2C). Examination of DAS markers at this time point (Figure 2B) shows a transient decrease in their expression (score 75% compared with 92% at passage 6) before returning to maximal levels. This time course corresponds with a transient change in the global expression profile of these cells observed between passages 6-8 in the original paper [26].

The dynamics of telomere shortening were not addressed in the original study. However, P7 occurs immediately before the onset of the main plateau phase of growth, suggesting that a significant fraction of cells may have undergone telomere dysfunction. DAS signalling spikes at P6, prior to the peak in mSS signalling at P7, raising the intriguing possibility that a threshold level of damage signalling triggers cells to communicate cellular stress to the surrounding microenvironment through the mSS pathway [40]. Notably, the spike in secretory signalling also correlates with a transient increase in expression of glycoprotein GPNMB/osteoactivin which was validated by QPCR in the original study [26]. GPNMB is an osteoclastic factor also implicated in endothelial cell adhesion and regulation of pro-inflammatory macrophage signalling, supporting the idea that signalling to the microenvironment is indeed increased at this time point [41,42]. To our knowledge this is the first time such a temporal switch in senescence signalling has been documented, however the differential regulation of secretory and other senescence pathways during replicative senescence is also concurrent with our other recent findings in human T-cells during senescence bypass [43].

### Senescence scoring in a Melanoma progression dataset

The above results suggest that the scoring system effectively captures increased activity of senescence pathways during both accelerated and physiological senescence. The expression levels of extant senescence pathways are likely to be altered by the processes of transformation and tumour progression and may reflect the route to immortalisation in individual tumour types. However, these questions have not yet been substantially investigated. We therefore examined the changes in senescence signalling during tumour progression in a melanoma dataset (GSE4587). Figure 2 shows levels of senescence signalling in benign nevi (score 55%, for all markers). Interestingly, an increased senescence score was found in melanoma when compared to benign nevi, regardless of the type of senescence signalling explored (DAS score: 58% versus 50%; mSS score 75% versus 63%). Although transformation has been achieved in these melanomas some aspects of the senescence program still appear to be active. The specific identities of induced senescence genes during tumour progression may point to the route of immortalisation for specific tumour types.

Comparison of primary melanoma and metastasis using all markers shows a slight increase in metastasis (melanoma score: 70%; metastasis score: 71.4%) (Figure 2A). However exploration of DAS and mSS reveals striking differences between the two tumour stages. Primary melanomas have a high contribution from mSS signalling and low levels of expression of the DAS biomarkers (75% and 58% respectively), while metastases have the opposite expression pattern (DAS score 75%; mSS score 69%) (Figure 2B and 2C). These results may suggest that the proinflammatory microenvironment produced by the secretory senescence signalling pathway is advantageous to a primary tumour in processes such as angiogensis. The low DAS senescence scores in primary tumours may reflect low levels of signalling shortly after immortalization and senescence bypass. After which, genomic instability may increase during the process of tumour progression leading to higher levels of DAS senescence signalling pathways in metastasis. Alternatively, DAS related signalling pathways may be induced during bypass of anoikis. In summary, these data suggest that DAS signalling increases during tumour progression. However these increases are clearly insufficient to induce cell cycle arrest.

### Correlation between senescence score and drug toxicity in the NCI60 panel

**Figure 3:**
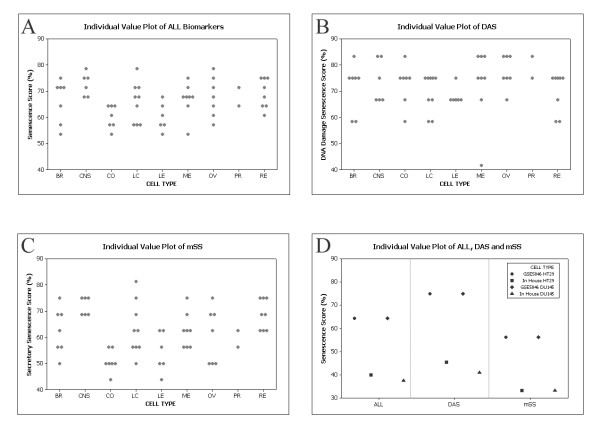
**Senescence scoring in the NCI60 cell panel**. Expression data from set GSE5846 were scored using (A) All markers from Table 1, (B) DAS markers, (C) mSS markers. Individual scores for each cell line are shown with cell lines separated according to tissue of origin. BR, breast cancer; CNS, central nervous system; CO, colorectal cancer; LC, lung cancer; LE, leukaemia; ME, melanoma; OV, ovarian cancer; PR, prostate cancer; RE, renal cancer. (D) Comparison of senescence scoring of public data for HT29 and DU145 cells with our experimental results.

The results above provide proof of concept that senescence scores obtained using these signatures conform with expectations given current understanding of replicative and accelerated senescence. To extend these results we investigated the possibility that latent senescence pathways may affect cellular responses to drugging. We first applied the scoring approach to gene expression data for the NCI60 cell line panel. Since extensive pharmacological characterisation data is available in addition to the expression profile in the dataset GSE5846 it is an ideal test set to examine the relationship between senescence signalling and drug response. Figure 3A-C show scores for all biomarkers (A), DAS (B) and mSS (C) in this panel with cell lines divided according to tissue of origin. For quality assurance purposes we also re-scored our own stock of two of the NCI60 cell lines, DU145 and HT29 and compared these results with those of the public dataset (figure 3D). Although we found somewhat lower scores overall, the trend toward higher expression of DAS than mSS is preserved in our hands in both lines (GSE5846 DAS scores: 75%, both cell lines; GSE5846 mSS scores: 56.3%, both cell lines; our cells DAS scores: 45.5% and 40.9%; our cells mSS scores: 33.3%, both lines).

Each tissue of origin had an individual scoring range profile. Comparison of scoring revealed clear divergence between DAS and mSS in some tissues. However in general DAS scores were higher than mSS. For example, leukaemia and colorectal cell lines generally scored more highly for expression of DAS markers than mSS (median scores: 66.7% (leukaemia), 75% (colorectal), DAS; 53.1% (leukaemia) and 50% (colorectal), mSS). Conversely CNS cell lines showed slightly higher mSS expression (mSS median score: 71.9%; DAS median score: 70.8%). These differences may reflect common routes to tumorigenesis and/or microenvironmental interactions in these tumour types. For example, increased secretion of pro-inflammatory and chemotactic molecules might be expected to select against blood-borne tumours whereas in solid tumours of immune privileged sites such as CNS, these may assist in processes such angiogenesis.

**Figure 4:**
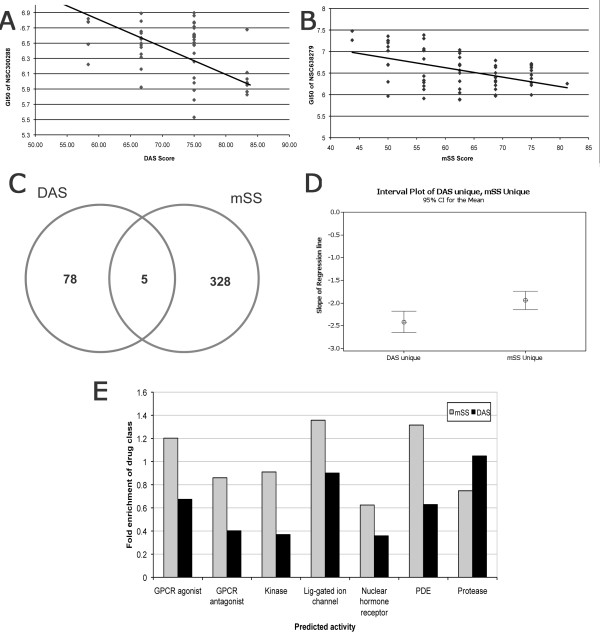
**Correlation between senescence scoring and cell line compound sensitivities**. Regression analysis was performed comparing DAS and mSS senescence scores derived from the NCI60 panel (figure 3, dataset GSE5846) with GI_50 _values for each cell line for ~1500 compounds reported in [44]. Representative regressions for DAS and compound NSC300288 (A) and mSS and compound NSC638279 (B) are shown. (C) Quantification of all compounds showing significant regression against DAS or mSS. (D) Characterisation of relationship between DAS/mSS and drug sensitivity. The slope of the regression for each significant DAS/mSS related compound was quantified. The figure shows the mean value across all significant compounds. (E) Activity modelling for significant compounds. Compounds were assigned predicted activity based on a decision tree model relating chemical similarity to a dataset of compounds with known activities. The proportion of compounds from DAS or mSS significant lists falling into each activity category is shown normalised to target class representation in the total compound set.

To explore the relationship between senescence signalling and drug induced toxicities in this panel, we analysed GI_50 _data for ~1500 compounds, tested at least four times against each of the cell lines, as described in a previous study by Scherf *et al *[44]. One finding presented in the original study, is a 1376 gene signature capable of clustering the cell lines according to their drug sensitivity patterns assessed by GI_50_. To determine if senescence signalling affects drug sensitivities, we performed regression analyses comparing the GI_50 _scores of each drug across the cell panel with senescence scores for DAS and mSS. Examples of significant regressions using the DAS and mSS signatures are shown in figures 4A and 4B, respectively, for compounds NSC300288 (p = 0.0015, slope = -3.34) and NSC638279 (p = 0.0002, slope = -4.04). Examination of regression trends across the compound set revealed 78 and 328 compounds with unique significant relationships (regression p < 0.05) between growth inhibition and DAS or mSS expression respectively, with a further 5 compounds displaying a relationship to both signatures (figure 4C). To explore the contribution of each signature to drug resistance or sensitivity we examined direction and slope of each unique significant regression for DAS or mSS (Figure 4D shows the mean value across all significant compounds unique for each signature). This analysis revealed that cell lines with high DAS and high mSS are generally more sensitive to compound induced growth inhibition, as shown by the overall negative regression slopes (average DAS slope = -2.41; average mSS slope = -1.93). These results suggest that latent expression levels of these pathways may potentially have a significant impact on response to some therapies.

### Compound activity prediction for significant DAS/mSS related compounds

To extend our analysis of the relation between DAS/mSS and drug sensitivity into prediction of target class sensitivities, we undertook activity modelling for the significant compounds using graph-theory connectivity indices to build a decision tree model based on chemical similarity with compounds of known activities in the classes GPCR agonist inhibition, GPCR antagonist inhibition, kinase inhibition, protease inhibition, PDE inhibition, ligand-gated ion channel inhibition and nuclear hormone receptor inhibition. We compared the fraction of assigned predicted activities for each target class in the DAS/mSS related compounds with those of the entire test compound set to obtain fold enrichment distributions as shown in figure 4E.

Protease inhibitor pharmacophores were enriched in the DAS related compounds compared with their representation in the test set (1.05-fold). Interestingly, since DAS apparently confers sensitivity to these compounds, these results may suggest that pharmacophores corresponding with existing protease targets may be therapeutically favourable in high DAS contexts. In all other drug categories mSS showed higher enrichment than DAS, where PDE inhibitor, Ion channel and GPCR agonist chemotypes were the most enriched in mSS related compounds (1.32-, 1.36- and 1.2-fold respectively). Since mSS also correlates with increased toxicity, intervention strategies based on such compounds may efficiently target mSS expressing tumours.

### Differential senescence signalling patterns in mesenchymal tumours

**Figure 5:**
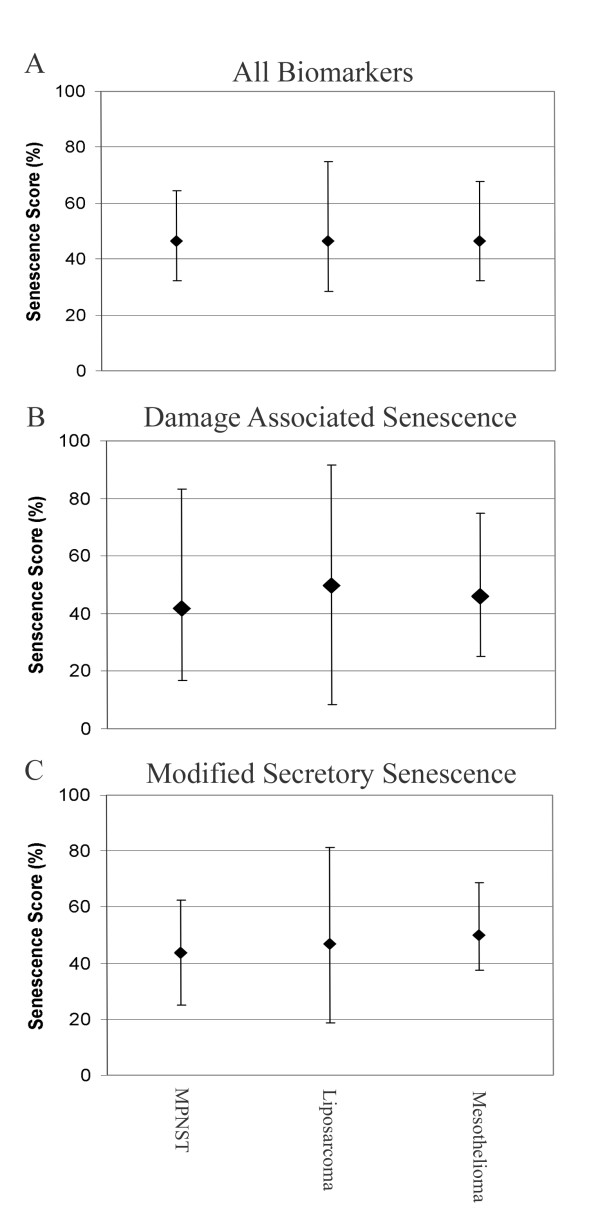
**Senescence scoring of mesenchymal tumours**. Peritoneal mesothelioma, liposarcoma and MPNST samples from set GSE17118 were scored using (A) All markers from Table 1, (B) DAS markers, (C) mSS markers. Median senescence scores are shown with bars representative of the maximum and minimum values.

Finally, to better understand the contribution of senescence signalling to the biology of mesenchymal tumours, we applied the approach to our own mesenchymally derived tumour gene expression dataset [28] (previously published under GSE17118 [45] (Figure 5). Considering all markers, the median scores were identical across all tumour types (46% (Figure 5A). Dissection of individual senescence signalling pathways showed that DAS signalling is lowest in MPNST (42%) and mesotheliomas (46%) and higher in liposarcoma (score: 50%) (Figure 5B). In contrast, mSS expression is higher in mesothelioma (score: 50%) than either MPNST or liposarcomas (44% and 47%, respectively) (Figure 5C).

These results suggest that even small changes in senescence signalling pathways can be detected using this scoring method and allow for hypothesis driven investigations. For example differences in extant/latent senescence signalling could affect factors such as patient prognosis and response to therapy.

We next investigated senescence score in individual tumour samples. For each senescence signature we ranked the scores for each tumour within its group to smooth the data. Ranked data was used to calculate correlation between senescence signatures for each individual tumour within the group (Table 2), allowing us to investigate whether the signatures are distinct signalling events or can be co-expressed in individual tumours. In all tumour types investigated both DAS and mSS signatures significantly correlate with All biomarkers (Table 2). Furthermore DAS and mSS never significantly correlate, suggesting that these two pathways are distinct signalling events at an individual tumour level. These data confirm that the results of the group analysis are pertinent at the level of individual tumours, implying that senescence phenotypes may be differentially activated during transformation.

**Table 2:** Correlation of senescence score rankings highlights signalling dynamics in individual tumours.

A MPNST	All	DAS	mSS
All	1		
DAS	0.738 (p = 0.001)	1	
mSS	0.792 (p =< 0.001)	0.245 (p = 0.361)	1

B Liposarcoma	All	DAS	mSS

All	1		
DAS	0.622 (p = 0.001)	1	
mSS	0.756 (p =< 0.001)	0.007 (p = 0.974)	1

C Mesothelioma	All	DAS	mSS

All	1		
DAS	0.858 (p =< 0.001)	1	
mSS	0.627 (p = 0.003)	0.164 (p = 0.489)	1

### Supervised Hierarchical clustering using the mSS signature highlights heterogeneous subgroups within mesenchymal tumours and a prognostic signature in peritoneal mesotheliomas

**Figure 6:**
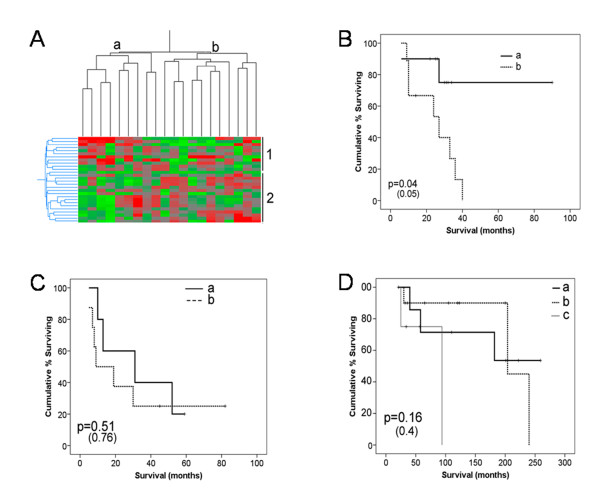
**Supervised hierarchical clustering using the mSS signature highlights gene groups correlating with survival in peritoneal mesotheliomas**. Clustering of mesothelioma samples is shown in (A). Group A tumours had higher expression of group 1 genes and lower expression of group 2 genes, corresponding to a potential pro-inflammatory phenotype. Gene groups are given in Table 3. (B-D) Kaplan-Meier survival analyses of tumour groups from mSS supervised hierarchical clustering. (B) Peritoneal mesotheliomas, (C) MPNST and (D) Liposarcoma. Only mesothelioma samples showed a significant association with survival improvement. P values shown in brackets are p-values after adjustment for age and sex. All survival analysis performed in SPSS (version 15).

Given the essential nature of the establishment of a secreted inflammatory network of signalling in senescence induction [46] and our observations of differential expression patterns of secretory senescence in mesenchymal tumours and hMSCs, we used the mSS signature to further explore senescence signalling patterns in this pathway in mesenchymal tumours. After normalisation of gene expression array data, we examined the expression patterns of only the mSS signature within each tumour type using supervised hierarchical clustering (Figure 6A, and data not shown).

Hierarchical clustering in peritoneal mesotheliomas split these tumours into two distinct groups, A and B (Figure 6A), corresponding to differential expression in two gene groups, 1 and 2 (Table 3). Specifically, group A tumours have higher expression of group 1 genes and lower expression of group 2 genes than do group B tumours. The groupings do not significantly correlate with any patients' characteristics for which we have data, such as age or sex (data not shown). Interestingly, tumours in group A are associated with improved survival compared to those in group B and this remains significant after adjusting for age and sex (Figure 6B). This suggests that even though senescence has been bypassed, the latent expression of different subsections of the secretory senescence pathway affects patient outcome.

**Table 3:** Gene groups responsible for clustering in peritoneal mesotheliomas.

Gene Group 1	Gene Group 2
CAMP	EEF1A1
EEF1A1	IGF1
IGF2R	IGF2
IL1A	IGFBP3
IL6	IGFBP5
IL8	IGFBP7
CXCR2	CXCR2
WNT2	PAI1
	STMN1
	TGFB1

Probes A_32_P47701, A_32_P44316 and A_32_P15320 for EEF1A1 appeared in group 1 while probe A_24_P763243 featured in group 2. Probe A_23_P135755 for CXCR2 appeared in group 1 while probe A_24_P933228 appeared in group 2.

Analysis of GO processes for these prognostic gene groups revealed that group 1 genes have functions in immune activation (p = 4.1E-10), chemotaxis (p = 1.6E-11), inflammation (p = 1.2E-8) and negative regulation of cell proliferation (p = 3E-16). This is concurrent with our previous findings in other tumour types, that patients with high levels of local inflammation and immune invasion at the tumour site have improved survival [47]. Furthermore, the genes in group 2 are involved with the regulation of cell growth (p = 3.4E-16), proliferation (p = 2E-13) and cell motion (p = 1.3E-14). The increased expression of genes involved in cellular proliferation and cell motion may be indicative of more aggressive tumours leading to decreased survival rate.

We performed the same analysis on the other two mesenchymal tumour types. Similar gene groups also split MPNST into 2 tumour groupings while liposarcomas were divided into three groups with high group 1 expression (A), low group 1 expression (B) and low expression of both groups (C) (data not shown). However, examination of survival revealed no significant difference between the groups in these tumours (Figures 6C and 6D). Nevertheless, these expression groups may reflect biological factors which might warrant further investigation to improve understanding of the underlying biology and role of senescence in these subgroups. The same analysis was also carried out using the DAS signature, however no clear groupings were produced by hierarchical clustering that would facilitate Kaplan-Meier survival analysis.

## Discussion

To improve the understanding of senescence signalling it is becoming increasingly necessary to look beyond the expression of individual genes and at the larger signalling processes they are involved in. Previous studies up to now have looked at senescence in terms of the expression of individual biomarkers [11,18,20], however we propose that senescence signalling may best be viewed as a network of gene interactions or indeed as a new gene ontology process as shown in Figure 1. Our senescence scoring approach allows us to look at senescence in a pathway directed way. By taking a percentage of the known senescence biomarker genes that are signalling in a pro- or anti-senescent manner we thereby reduce the information given by multiple biomarkers down to one quantifiable number. It is then possible to dissect the levels of particular senescence signalling pathways occurring in a sample at any one time and directly compare the levels of these pathways, giving us an insight into subsystems involved in senescence establishment and maintenance. In this study we have validated the approach using a number of publicly available datasets corresponding to scenarios where modulation of senescence signalling might be expected. We also performed an in-depth study into secretory senescence in our own mesenchymally derived tumour datasets. We have shown that senescence signalling occurs in a context dependent manner. In certain situations DAS and mSS programs coexist, while in others they are distinct signalling events regulated in a time dependent fashion.

The induction of DNA damage by radiotherapy in cancer is commonplace in current treatment regimes [34]. Recent evidence suggests that senescence is frequently induced after radiation exposure [4,48,49]. Through the examination of colon tumours and normal tissues pre and post radiotherapy from publicly available data, we aimed to gain a further understanding of the individual roles of DAS and mSS signalling in this process. As well as observing a general increase in senescence signalling post radiation exposure concurrent with the literature, we have shown that secretory senescence signalling is not induced by radiotherapy in colon adenocarcinoma, while there is a trend in favour of an increase in DAS signalling in these samples. To our knowledge, this distinction has not previously been made. The scoring method allows us to measure senescence and to form hypotheses on the effect of modulating the response, for example lower mSS signalling in adenocarcinoma post-radiotherapy may present important opportunities for immune activating adjuvant therapies through the activation of secretory senescence signalling in tumours of patients receiving radiotherapy. In addition the observation that cancer cells express senescence markers, but are able to continue proliferating suggests that there is a threshold level of expression for senescence to occur or that key effector molecules are not present. At present this threshold level is unknown, however the differential levels of senescence seen in tumours compared to normal tissues could suggest that tumours are closer to senescing than their normal counterparts and therefore may signify a therapeutic window, which could be exploited. These hypotheses can now be tested experimentally.

We next applied the scoring to a gene expression dataset comprising doxorubicin or 5-FU treatments of the breast cancer cell lines ZR-75-1, ME16C, and MCF7 [25]. Here again we found increased representation of all biomarkers, consistent with the emerging concept of drug-induced accelerated senescence. The scoring system therefore also reported increased senescence in a second therapeutic intervention scenario. In contrast with the radiotherapeutic context, both DAS and mSS subsystems were induced by drug treatments although induced DAS signalling still predominated. The distinction between the levels of mSS and DAS in response to the two treatment types, to our knowledge, has not previously been documented.

To determine whether the scoring system identified increased senescence signalling in the normal "physiological" context of replicative senescence, we investigated a time-course dataset corresponding to gradual replicative senescence of hMSCs with increasing passage [26]. Investigation of all biomarkers showed a gradual increase with passage with a plateau between passages 6-8. By dissection of DAS and mSS elements we found that this plateau corresponded to a point at which DAS signalling sharply increased. This was subsequently followed by an increase in secretory senescence signalling concurrent with a transient decrease in DAS signalling. Such a decrease in DAS signalling may highlight a specific time-point for the secretory senescence pathway to signal cellular distress to surrounding cells, as previously observed in the literature [40] and further highlights the distinct nature of the two signalling pathways. To our knowledge this is the first time such a temporal switch in senescence signalling has been documented.

Previous data on individual senescence genes in melanoma has suggested that senescence is a barrier to tumour progression [50]. Application of the scoring approach to a melanoma progression dataset showed that not only did senescence signalling remain active after immortalization but that the levels of senescence signalling of all types in primary tumours were higher than that seen in benign nevi. This suggests that although the senescence program has been bypassed the signalling pathways continue to operate in melanoma. Furthermore, we found differential expression of secretory and DNA damage/chromatin senescence pathways in primary lesions and metastasis, with secretory elements of senescence showing a trend towards down-regulation in metastases, which may facilitate immune evasion and disease progression. Despite the down-regulation of secretory senescence the DAS elements of senescence continue to signal. The latent signalling of senescence in tumours and metastasis may present opportunities for therapies to reinstate the correct endpoint of these pathways and halt further disease progression. Our study therefore suggests that therapies targeted to induce secretory senescence in metastatic melanoma may warrant further investigation and together, the results from these public datasets confirmed that the scoring system is able to detect the contribution of individual senescence signalling subsystems in a variety of contexts.

We therefore investigated a possible relation between senescence score and compound sensitivities in the NCI60 panel using growth inhibition data from ~1500 compounds reported in [44]. By regression analysis we found a subset of compounds for which GI_50 _across the cell panel significantly correlated with DAS or mSS score. Combining all significant results for either signature, we found an overall negative correlation between DAS and mSS score and GI_50_, indicating that high DAS and mSS score may confer sensitivity to drugging. Modelling of compound activities revealed overrepresentation of protease inhibitor like pharmacophores in the DAS subset and ion channel/PDE inhibitor and GPCR agonist like structures in the mSS subset. These results indicate that senescence scores may yield predictive information on cellular therapeutic sensitivities.

We next performed a more in-depth investigation of senescence signalling in individual mesenchymally derived tumours. Initial examination of the correlations between the rankings of individual tumours from 3 main mesenchymal malignancies, liposarcoma, MPNST and mesothelioma, highlighted similar patterns of senescence signalling in individual tumours of all 3 tumour types. Scores for All biomarkers significantly correlated with those for DAS and mSS in all cases. In contrast DAS and mSS scores show no correlation suggesting that these signalling processes are distinct events at an individual tumour level. This is consistent with analysis of the larger tumour groups, where we observed increased mSS scores in mesothelioma and increased DAS in liposarcoma in comparison to the other tumour types. As distinct events we hypothesise that these signalling processes may impact upon other clinical factors such as prognosis or response to treatment.

Previous studies have examined individual senescence markers and their prognostic significance in these tumour types [29-31] but not the prognostic significance of entire senescence signalling pathways. Distinctions between the different senescence signalling dynamics in individual tumours gives improved understanding of the cellular context prior to treatment being applied and potentially helps to make the move towards patient tailored targeted therapeutics more realistic. Given the importance of the establishment of a secretory senescence network in the induction of senescence [46] we hypothesised that this may be a pathway that could be perturbed by different mechanisms to effectively prevent senescence induction. We therefore undertook a more in-depth analysis of the patterns of expression of secretory senescence in the three mesenchymal tumour types.

Hierarchical clustering of peritoneal mesotheliomas split the tumours into two groups based on distinct expression patterns of subsets of secretory senescence genes involved either in proinflammatory and immune activating processes or in pro-growth and proliferation processes. Interestingly, the groupings showed significant correlation with survival; in particular, those tumours displaying increased expression of pro-inflammatory genes had improved survival compared with the lower expressing group. The functions of these genes are therefore likely to be of importance in the underlying tumour biology of mesotheliomas. Although hierarchical clustering also divided the MPNST and liposarcoma samples into 2 and 3 clusters, respectively, based on similar gene groups, no association with survival was observed. However, we have previously shown improved survival associated with tumours with increased immune infiltration [47] and the activation of specific secretory senescence pathways may facilitate this. Given the secretory nature of these molecules, their measurement in patient serum will improve understanding of the role of senescence signalling in patient outcome for these and other tumour types.

## Conclusions

The application of senescence scoring is a useful tool for assessing senescence signalling in general, as well as individual senescence pathways, applicable to any gene expression microarray dataset. The flexibility of this method facilitates its application to other biological processes or modulation of signature content as knowledge in the field progresses. Application to other tumour types could also significantly improve our understanding of senescence signalling in cancer and aid in the development of mechanistic studies of these pathways. This initial study of latent senescence signalling in human tumours provides a first-glimpse of its potential importance in tumour biology.

## Methods

### Senescence scoring of gene expression data

Publicly available or in-house generated gene expression microarray data files were imported into BRB ArrayTools (BRB-ArrayTools developed by Dr. Richard Simon and BRB-ArrayTools Development Team [33]) and filtered to show only values associated with the senescence signature gene list in Table 1. Filtered data was then exported to Microsoft Excel (Microsoft Corp.) for further analysis. Data was normalised to a median value calculated across all arrays in that experiment so that data >1 was up-regulated and <1 down-regulated relative to a median of 1. Genes were scored as "senescent" or "non-senescent" depending on their gene expression levels relative to the median and their known expression pattern during senescence signalling (see Table 1). For example, expression of the proliferation-associated gene *KI67 *below median would be marked "senescent" but above median expression would not; likewise expression of the cyclin dependent kinase inhibitor p21 above median would be marked "senescent" but below median expression would not. The number of genes signalling in a "senescent" direction was then summed and represented as a percentage of the total number of genes expressed from the senescence signature. For example, when 20 of the 28 genes in Table 1 were expressed and 16 genes were signalling in a pro-senescent manner the score was 80%.

To calculate DAS and mSS scores data was sorted in Microsoft Excel and split into the two signature lists. Scores were calculated as a percentage of only DAS or mSS expressed genes respectively.

Median, maximum and minimum values were plotted using Microsoft Excel or Minitab (v15, Minitab Inc, Coventry, UK).

### Correlations of senescence signature rankings

To assess the correlations of senescence scores given to each individual mesenchymal tumour by each signature the scores for each tumour were placed into Minitab (v15, Minitab Inc, Coventry, UK) and rankings for each signature score and Pearson correlations calculated. Regression analysis comparing NCI60 GI_50 _data with senescence score was performed using the statistical package of Perl and exporting results to Excel.

### Chemoinformatic prediction of compound activities

The dataset was pre-processed to remove couterions, assign physiological pH-appropriate atomic charges and add hydrogen atoms. Fifty 2D molecular descriptors were computed for each compound including Petitjean graph theory descriptors, Weiner path and polarity descriptors, GCUT descriptors, Kier and Hall connectivity indices, Zagreb index, Balaban indices, Van der Waals surface area and volume descriptors and topological polar surface area [51]. The descriptors were also computed for a training dataset of 3,537 publicly-available known active compounds collated from the PubChem database and the ChEMBL database (437 kinase inhibitors, 781 protease inhibitors, 806 GPCR agonists, 737 GPCR antagonists, 100 PDE inhibitors, 297 nuclear hormone receptor inhibitors and 379 ligand-gated ion channel inhibitors). This database of known actives was input to a binary decision tree algorithm, to derive a 7-category decision tree using 38/50 descriptors which correctly classified 62% of the dataset. The decision tree was subsequently used to predict activities of the NCI60 test dataset. All modelling conducted within the MOE software suite (Chemical Computing Group Inc, 1255 University Street, Montreal, Quebec, Canada).

### RNA extraction and Gene expression microarray analysis of Mesenchymal tumours

A subset of 24 liposarcoma samples and 20 peritoneal mesotheliomas from a larger study population previously described [52,53] and 16 MPNST were used for gene expression analysis. All samples were obtained under local ethical approval. RNA extraction and microarray hybridisation was performed as described in [28,52,53]. Fully MIAME compliant data files have previously been published under accession number GSE17118 [45].

### Hierarchical Clustering and Survival Analysis

Hierarchical clustering of each tumour type was performed using Spearman correlation, merging branches with similarity less than 0.01 in Genespring GX (v7.3). Kaplan-Meier plots of survival and Log Rank (Cox Regression) tests adjusting for age and sex were performed on all resulting first level clusters of samples in all 3 tumour types using SPSS (v15, SPSS Inc, Chicago, Illinois, USA).

## Authors' contributions

WNK conceived and supervised the study. KLW, NBJ, KAO and WNK participated in study design, CJC, AB, KLW, NZ, SBurns, SBoyd, JR and LH performed data analysis and evaluated results. All authors have read and approved the final manuscript.
